# “Knowing It Before Blocking It,” the ABCD of the Peripheral Nerves: Part D (Approach to the Patient With Nerve Injuries)

**DOI:** 10.7759/cureus.41782

**Published:** 2023-07-12

**Authors:** Kartik Sonawane, Hrudini Dixit, Navya Thota, Aparna Jayaraj, Jagannathan Balavenkatasubramanian

**Affiliations:** 1 Anesthesiology, Ganga Medical Centre and Hospitals Pvt. Ltd., Coimbatore, IND; 2 Anesthesiology, Sir H. N. Reliance Foundation Hospital and Research Centre, Mumbai, IND

**Keywords:** treatment of nerve lesions, management of nerve injuries, neurological damage, pain management, nerve injury

## Abstract

“Prevention is always better than cure.” However, despite all precautions or preventive measures, sometimes patients develop neurodeficits due to suspected nerve injury in the perioperative period. Assessment and evaluation of the patient’s symptoms can provide clues to the causative factors. Such causative factors can be corrected immediately to avoid further deterioration, or some may require further workup. The management plan for such a diagnosed nerve injury depends on the symptoms, the finding of the medical history, and the diagnostic imaging and tests. Simultaneous symptomatic relief in the form of pain medications, steroids, anti-inflammatory drugs, psychological counseling, and reassurance is essential to expedite treatment goals.

Diagnosing and treating nerve injuries cannot be laid down as a straightforward part. It is a zigzag puzzle in its own right, playing with time and injury progression. Careful assessment to diagnose the extent of nerve damage plays an important role in treatment plans. It helps decide when to proceed and when to postpone, whether conservative strategies would suffice, or surgical repair would be required. Although most nerve injuries are self-limiting, some cases require surgical intervention that needs to be diagnosed early. The revolution was started by Sunderland in 1945 when he described neurosurgical techniques that drastically changed the entire scenario of nerve repairs. The ultimate effective treatment and full recovery may not be guaranteed, but attempts must be made to achieve the best results. With the patient’s interests in mind, it is important to formulate a plan ensuring a good quality of life with minimal impact on their daily activities. Multifactorial nerve injury requires a multidisciplinary approach that primarily includes reassuring, psychological counseling, multimodal analgesia, and neurological and occupational consultations.

This article describes the step-by-step approach known as the symptoms categorization-history taking-examination-diagnostic evaluations (SHED) approach to managing patients with peripheral nerve injuries. It also details the various modalities for diagnosing nerve injuries, sequential electrodiagnostic studies, and treatment plans depending on the type and extent of nerve injuries. It will help readers to design a treatment plan based on the patient’s symptoms and evaluation results.

## Introduction and background

Despite all precautions or preventive measures, sometimes a patient can develop neurodeficit due to suspected nerve injury in the perioperative period. What has been done cannot be undone but can be prevented from happening again. Reassuringly, most postoperative sensory changes occur within the first week of injury, 95% resolve within four to six weeks, and 99% resolve within the first year [1-3]. However, managing patients with nerve injuries raises many questions: What might have happened? Where did things go wrong? Was that avoidable? How to justify it and defend it in a court of law? Answers to all such questions lie in the detailed understanding of specific technicalities, minutiae of nerves (anatomy and physiology), pathology associated with nerve injuries, risk factors, and preventive measures to avoid them. A stepwise approach to managing patients with nerve injury can be essential to help identify, evaluate, diagnose, and finally decide the treatment plan. Identifying the correctable causes and the acute progressive causes is very important to avoid further deterioration. Since the type of nerve injury determines the prognosis and further treatment plan, the identification, localization, and subsequent categorization of nerve lesions is crucial in the diagnostic assessment.

Diagnosing and managing nerve injury is equally important to avoid increased hospital stays, reduced patient satisfaction scores, fear or noncompliance to future regional anesthesia (RA) techniques, and medicolegal issues. Fortunately, most neurological damage that occurs during peripheral nerve block (PNB) usually resolves spontaneously. Any neurological deficit, sensory or motor, beyond the block duration is unexpected and undesirable, which can be very distressing for the patient. Therefore, reassurance and good communication are important when dealing with such circumstances and their medicolegal perspective. A concurrent focused clinical assessment is necessary to identify the evolving processes (such as compartment syndromes or hematoma formation) or the correctable/repairable factors (like transection of nerve or important vessels) to avoid further damage.

This article is part of the comprehensive overview of the essential understanding of peripheral nerves before blocking them. It describes a stepwise approach to the patient with suspected nerve injuries. It helps readers in further planning to identify and diagnose the type of lesion and establish a treatment plan according to the type and severity of the nerve lesion.

## Review

This narrative overview describes a stepwise approach to the patient with suspected nerve injuries, symptomatology, diagnosis, and management of nerve injuries. Related literature searches were performed using online platforms (PubMed, Medline and Embase databases, Cochrane Library, and Google Scholar) using relevant search terms (neurons/nerve damage/nerve injuries symptomatology/management of nerve injuries/treatment of neuropathic pain/electrophysiological studies/multimodal analgesia in nerve injuries). Articles published in English were selected, and their reference sections were manually searched for additional information.

Initial assessment and evaluation

Early diagnosis of postoperative peripheral nerve injuries (PNIs) can be difficult due to certain obstacles in assessing and recognizing symptoms. These include residual sedation, the effects of a PNB, pain (limiting examination), casts, bandages, splints, slings, and restricted range of motion. Sometimes the patient does not share the concerns, assuming the neurological deficit after PNB is normal even if it persists beyond the block duration. The neurodeficits (Table 1) due to suspected perioperative nerve damage can be recognized based on their typical characteristics like onset, symptoms, location, and progression [4-6]. However, important aspects of the initial assessment also include identifying potential risk factors and recognizing neurological symptoms associated with nerve injury.

**Table 1:** Characteristics of neurodeficits associated with perioperative nerve injuries

Characteristics of neurodeficits due to perioperative nerve injury	
Onset	Beyond block duration.
	>48 hours.
Symptoms	Mild tingling.
	New onset of pain.
	Paresthesia.
	Numbness.
	Severe sensory-motor deficit.
Location	Nerve block territory.
	Common entrapment sites.
	Global.
	Multifocal.
	Diffuse.
Progression	Acute progressive symptoms need to be addressed promptly.
	Nonprogressive symptoms need further workup.

Early involvement of the surgical team is advisable in cases of suspected neuropathy possibly caused by prolonged tourniquets, casts, excessive intraoperative traction, or improperly placed surgical clips. Existing motor deficits and developing or severe/complete neuropathies require consultation with a neurologist and/or neurosurgeon. Diagnostic testing is recommended when symptoms are not purely sensory or neuropathy is severe and/or persistent.

“SHED” approach

**Figure 1:**
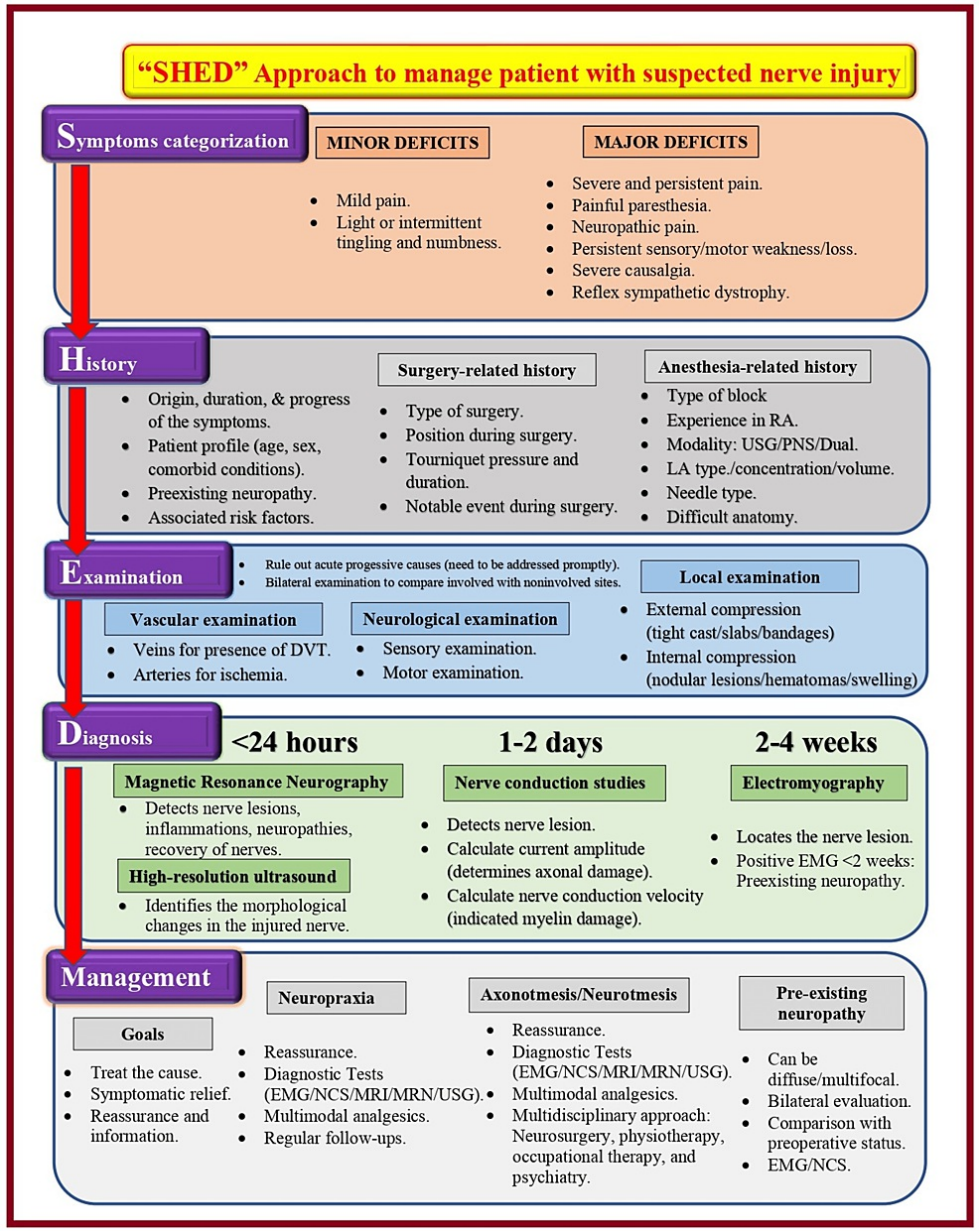
“SHED” approach to manage patients with nerve injuries RA: regional anesthesia; USG: ultrasound guidance; PNS: peripheral nerve stimulator; DVT: deep venous thrombosis; EMG: electromyography; NCS: nerve conduction; MRN: magnetic resonance neurography Source: This figure was created by the first author (KS).

Designing a treatment plan based on the patient’s symptoms requires a step-by-step approach. The SHED (Symptoms categorization-History taking-Examination-Diagnostic evaluations) approach (Figure 1) can be helpful when approaching nerve injury patients. It incorporates symptom categorization, history taking, physical examination, and diagnostic measures for definitive diagnosis.

Symptoms Categorization

Nerve injuries can manifest as pain, paresthesia, and sensory and motor deficits. The sensory and motor deficits may be newly developing or the advancement of pre-existing neurological findings. Symptoms of nerve injury usually appear after the expected duration of PNB. This duration also depends on the local anesthetic (LA) agent (type, concentration, and volume), adjuvant type, and pre-existing neuropathy. The duration and intensity of nerve injury symptoms depend primarily on the severity of the nerve lesion. Neurological symptoms can be considered permanent if they persist for over 12 months. Some factors can influence postoperative symptoms associated with nerve injury, including surgery-related pain, immobility, the position of the patient, and the application of casts, dressings, bandages, or splints. Neurological symptoms following nerve injury can be divided into major and minor deficits based on the nature, duration, and severity. Scrutiny of each symptom via history-taking will help to make further progress.

History Taking

“Always listen to the patient; they might be telling you the diagnosis” (Sir William Osler, 1849-1919). History-taking and empathetic communication are key steps to clinical diagnosis and the doctor-patient relationships while managing a patient with a nerve injury. It helps alleviate patients’ anxiety about existing symptoms, builds rapport and trust, and enhances treatment compliance. It helps to establish a link between presenting symptoms and possible causative factors. It should focus on finding associated risk factors, pre-existing neuropathy (such as diabetic or thyroid neuropathy), origin, duration, progression of symptoms, and changes in symptom severity. Simultaneous analysis of clinical documents helps to understand the causative factors of surgery and anesthesia.

A properly collected history can reveal the patient’s beliefs, concerns, expectations, and key accompanying diagnoses. It also provides clues to suspected causes of a PNI, whether it is acutely progressive or related to common entrapment sites, PNB/anesthesia, surgical causes, or pre-existing neuropathy. Proposed diagnoses can be confirmed with further steps, including a physical examination and diagnostic evaluations.

Physical Examination

The physical examination aims to rule out acutely progressive causes of PNI from nonprogressive causes. The acute progressive causes must be addressed urgently to prevent further deterioration. However, the nonprogressive causes need further investigation to diagnose the type and severity of the nerve injury. The physical examination should primarily focus on the vasculatures to rule out vascular pathologies, the nervous system to assess sensory/motor functions and reflexes, and local examination of the surgical site and block site to rule out local causative factors. Bilateral physical examination helps compare affected and unaffected sites, which can provide information about the degree of pathology.

Vascular system: A vasculature examination is performed to rule out injury to arteries, veins, or both. It also reveals signs of deep venous thrombosis, including skin color changes, engorgement of veins, edema formation, hematoma, or pain at the surgical or block site. Injury to the arteries is mainly associated with the rapid onset of intense pain over the affected area and sometimes paresthesia and cold [7,8]. The arterial injury can require an emergent surgical exploration. Injuries to the veins are mainly associated with edema formation and engorgement of veins distal to the lesion [7,8]. Deep venous thrombosis is common in patients with lower limb joint replacement surgeries or those who are immobile for longer (>72 hours) [9]. The pain is mostly in the back of the leg (sciatic distribution) and is sometimes associated with leg swelling [10]. This type of pain is typically not relieved by the femoral nerve block alone. The presence of tight casts, dressings, and bandages should be evaluated, which can cause vascular compromise. The diagnostic test to confirm the findings of vascular examination includes a vascular Doppler study or computerized tomography (CT) angiography [11].

Nervous system: A thorough neurological examination should be initiated after ruling out vascular involvement causing nerve injury. It includes sensory and motor examination and assessment of the reflexes. Newly occurring sensory loss or alternation, muscle weakness, or changes in reflexes can be identified during the neurological evaluation. The knowledge of the involved dermatomes and myotomes can be used to localize the impairment of the potential single peripheral nerve or plexus [12]. Different types of sensations are transmitted to the brain via different sensory pathways. Therefore, specific patterns of sensory impairment can indicate the location or type of injury. During the sensory examination, the skin surface of the affected and unaffected sites should be systematically tested to determine any pattern of the deficit [13]. Light stimulus in the form of cotton swabs or light finger touch can be used to test light touch sensation. Sharp pain sensation can be tested using the pinprick end of the safety pin or hypodermic needle, whereas the dull pain sensation can be tested using the rounded/dull portion of the pin. Joint position sense can be tested by asking the patient about the changed position of particular distal joints of extremities with closed eyes. Temperature and vibration sensations can also be tested using a tuning fork.

Motor assessment [13] can be made through observation, inspection, and palpation of visible abnormalities and functional muscle tone and strength testing. Every precaution should be taken to avoid exacerbating certain deficits during neurological examination. Any unusual symptoms such as muscle twitching, tremors, involuntary movements, or tenderness should be pointed out. The deep tendon reflexes should be tested using a percussion hammer in order to test the functional sensory and motor fibers of a respective spinal level. Reflex responses can be categorized as normal, hyporeflexia, or hyperreflexia. The bilateral (right and left) reflex responses should be compared and checked for symmetry. Commonly tested reflexes with associated spinal levels include biceps (C5/C6), triceps (C7), patellar (L2/3/4), and ankle (S1).

Local examination: A local examination of the PNB, common entrapment, tourniquet, and surgical sites can help rule out possible factors that can cause external or internal compression on the neurological or vascular structures. Such examination also helps to rule out an ongoing increase in compartment pressure. Neurological deficit distal to the surgical site can indicate surgery-related causes. Similarly, block-related, position-related, or tourniquet-related causes also can be determined on thorough local examination.

Diagnostic Evaluations for Nerve Injuries

Nerve injury is always a serious concern for the patient, surgeon, and anesthetist. It leads to many questions that must be answered: Is there an actual nerve lesion or not? If yes, where exactly is it located? What type of lesion is it? How much time will it take to recover? Who can be held responsible for it (the patient, the operating surgeon, the anesthetist, or surgery-related factors)? Answers to such questions can be obtained from available diagnostic tests for nerve injury in addition to presenting symptoms, history, and examination.

Mild deficit symptoms usually subside within a few days or weeks without any need for further diagnostic evaluations. Major deficit symptoms are persistent and severe, commonly associated with intense pain requiring immediate neurologist/neurosurgeon consultations and supportive care like reassurance, multimodal analgesia (MMA), and further diagnostic testing. Diagnosing and treating nerve injuries cannot be laid down as a straightforward part. It is a zigzag puzzle in its own right, playing with time and injury progression. Among the various nerve injury causes, some require urgent intervention to prevent further damage. Therefore, the timing of considering the patient for diagnostic testing is very critical. With a thorough understanding of the symptomatology, history, and physical examination findings, those causes of nerve injury that require urgent surgical intervention should be identified first. For example, causes such as a hematoma requiring urgent evacuation or compartment syndrome requiring urgent fasciotomy to prevent further deterioration. Diagnostic evaluations can be ordered for other nonprogressive causes of nerve injury to determine prognostication and diagnosis of the type of nerve injury.

The choice of diagnostic tests depends on the time since nerve injury and the time required to complete post-injury physiological processes (like degeneration, regeneration, remyelination, and reinnervation) in the nerve (described in Part B of this review). Available diagnostic tools for PNI include high-resolution ultrasound, magnetic resonance imaging/neurography (MRI/MRN), computerized tomography (CT) scan, and electrophysiological testing.

High-resolution ultrasound

**Figure 2:**
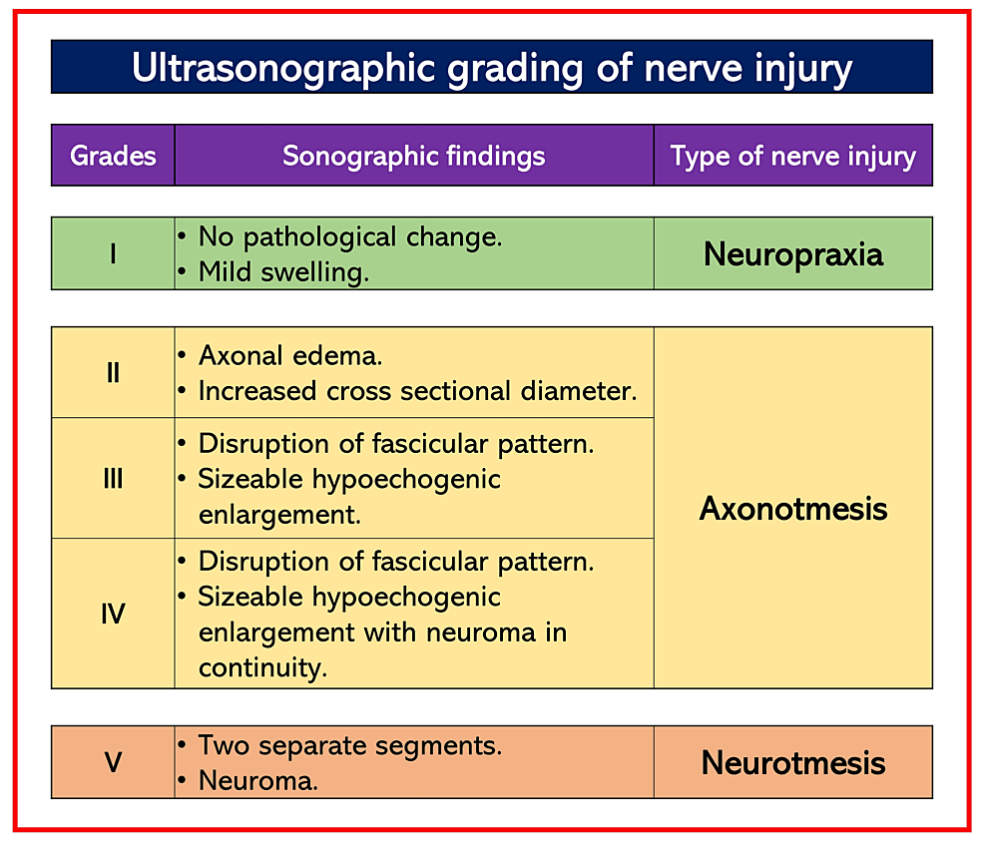
Ultrasonic gradings of nerve injury Source: This figure was created by the first author (KS).

Using ultrasound in RA practice helps ensure safe RA by avoiding injury to vital structures such as nerves or vessels. It also helps diagnose nerve injury and its progression along with pathologies in the vessels, muscles, and soft tissues around neural structures. The severity and extent of nerve lesions can be graded (Figure 2) based on the type of nerve injury using an ultrasound scan [14-16]. The ultrasound helps to gain structural information from the vessels of the upper and lower extremities. A modern, high-resolution, ultra-high-frequency ultrasound machine can even detect morphological changes in the peripheral nerve after nerve injury. It can detect neuromas, hematomas (precise location and extent), fascicular disruption, identification of small nerves, and nerve enlargement in the early stages of neuropathy [15,17]. It provides better demarcation and delineation of the nerve ultrastructure. It can even detect real-time intraneuronal injections. The intraneuronal injection can be diagnosed when the nerve area increases by >/=15% and one or more additional ultrasound markers (such as nerve swelling or proximal-distal diffusion within the epineural tissue) are present [18]. However, even 0.1 ml of injectate is sufficient to rupture a fascicle. High frequencies like 15-18 MHz for superficial nerves (median) and 9-12 MHz for deep nerves (sciatic) are recommended [19].

Ultrasound also helps screen and diagnose deep vein thrombosis, pre-existing neuropathy, acute nerve injury, and nerve regeneration. Therefore, it is an important tool for initial imaging to localize nerve lesions in suspected PNI or subsequent cases. It is a faster diagnostic tool that is more cost-effective than MRI. Unlike MRI or CT, it can also be used as a bedside diagnostic tool. However, it requires an experienced and skilled operator to visualize and interpret images. Also, high-frequency ultrasound can only obtain superficial images up to 2 cm below the skin.

**Figure 3:**
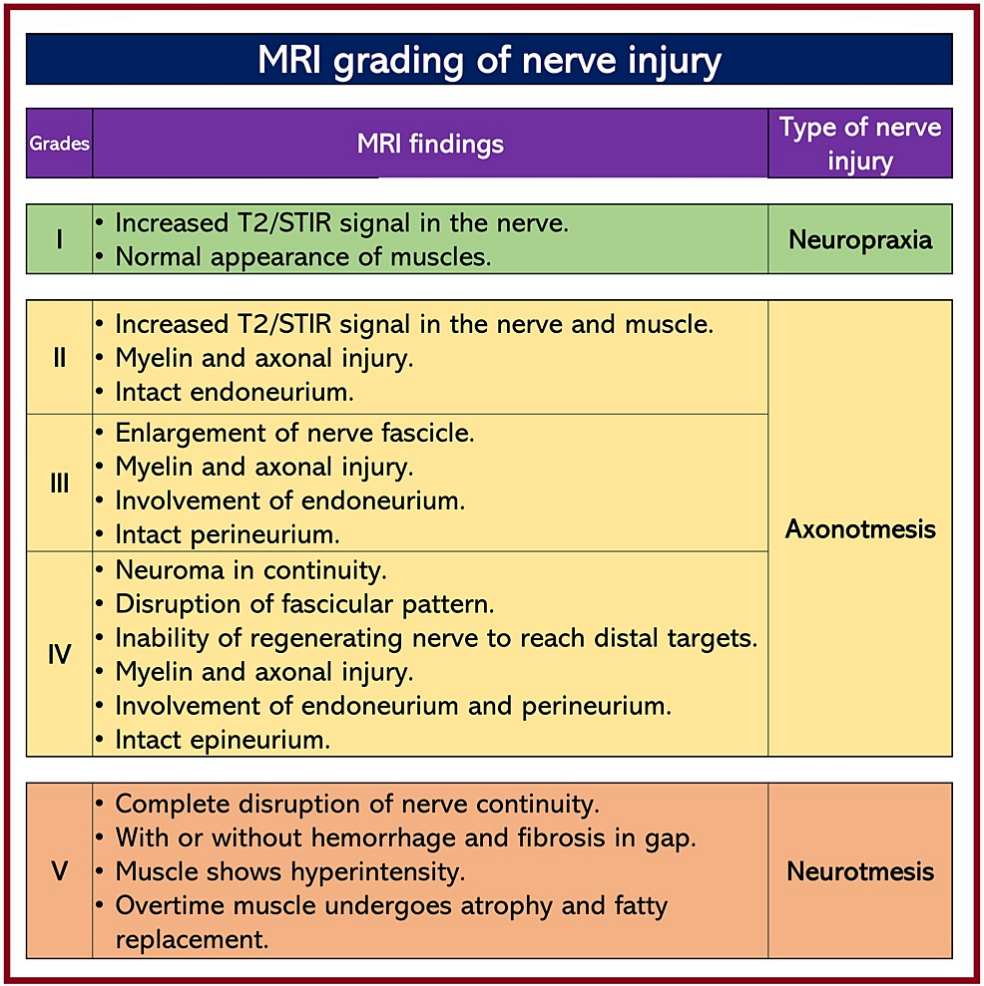
Nerve injury gradings based on magnetic resonance imaging/neurography (MRI/MRN) MRI: magnetic resonance imaging; STIR: short tau inversion recovery. Source: This figure was created by the first author (KS).

MRN: MRN, a high-resolution MRI, is a new imaging method for displaying peripheral nerves, in which three-dimensional images of the nerves can be obtained. It can provide high-quality information about nerve lesions, inflammation, neuropathy, and nerve repair. Like ultrasound, MRN can also be used to grade (Figure 3) nerve injury types [20-23]. It is the earliest form of modality to detect nerve injury, which can detect an axonal nerve lesion as early as 24 hours after injury. However, it requires an experienced operator and has limited access to patients. MRN is considered a highly sensitive modality, but its specificity for neuropathies is still in doubt [24,25]. It is coupled with problems such as huge costs, long scan times, and device availability. Like MRN, MRI can also provide better soft tissue delineation and localize the injury, edema, or hematoma.

CT scan:** **A CT scan can be used when an MRI facility is unavailable, or the patient needs to be transferred for it. It can be considered the first line of imaging to avoid such delays. However, it can only give a rough idea of whether there is edema, compression, fibrous tissue, or a lesion near the nerve. In addition, a negative CT does not exclude the possibility of nerve injury. Therefore, MRI should always be performed after a negative CT to confirm the presence of nerve injury [26].

Electrophysiological testing: Electrophysiological testing can be used as an important diagnostic tool that can help clear doubts by identifying and locating nerve lesions. Such testing plays an important role as an extension of the clinical examination in patients with nerve injuries. It helps identify the lesions through nerve conduction velocity (NCV) studies and locating them through electromyography (EMG). In addition, it provides information about the involved nerve fiber types and helps determine the type, severity, and prognosis of the nerve injury [27,28]. Therefore, electrophysiological studies are the mainstay for localization, severity, and prognostication for various types of PNIs, not only the injury caused by PNB.

NCV/nerve conduction studies (NCS): NCV or NCS includes motor conduction studies (MCS) and sensory conduction studies (SCS). It is performed by placing two cutaneous electrodes (stimulating electrode and recording electrode) along the path of the nerve. The current is delivered through a stimulating electrode, and the conduction of current in the form of a specific wave pattern is recorded through the recording electrode. The response is recorded either from muscle or from the nerve. The response obtained from the muscle is called compound muscle action potential (CMAP) [28], which is the summation of all motor unit potentials (MUP) under the electrode. The sensory response obtained from the nerve is called sensory nerve action potential (SNAP) [28].

Parameters Measured

Amplitude: It corresponds to the strength of the current used to stimulate the nerve or height of the action potential. It is proportional to the number of axons stimulated. The amplitude of SNAPs is usually between 5-20 μV. In comparison, the amplitude of CMAP is about 100 times larger than SNAP [29]. The reduction in amplitude indicates axonal damage. A reduction in amplitude to 50%-70% suggests a conduction block [30].

Latency: It is the duration from delivering threshold current to eliciting response or action potential.

Conduction velocity: It is the speed at which the action potential or impulse travels. It is calculated as the ratio of the distance between the placed electrodes and the time required for signal transmission. It represents myelination and axonal integrity. The decrease in conduction velocity indicates myelin damage.

Duration: It is the time gap between the onset and the termination of latency.

Timing of NCS

NCS can detect the nerve lesion within one to two days of injury [31,32]. Therefore, the initial NCS performed only after one to two days of injury. Despite an initially normal NCS study, it can be repeated in 10-14 days if the symptoms persist [33]. Subsequent NCS determines the prognosis of the detected nerve lesion.

Results

Normal NCS: The presence of response (CMAP/SNAP) with normal conduction velocity indicates the absence of a nerve lesion. A response with decreased conduction velocity indicates a neuropraxia type of nerve lesion. The NCS can also be normal if the lesion involved unmyelinated nerve fibers responsible for pain during the study.

Abnormal NCS: Abnormal NCS or absence of response indicates the presence of nerve lesions that can be located further by EMG studies.

Limitations

The conduction along the nerve fibers depends on the diameter and presence/absence of myelin sheath. Due to high conduction velocity, the NCS electrode records signals only from myelinated axons. In comparison, signals from unmyelinated axons remain ignored.

Electromyography

EMG study locates the nerve lesion identified by the initial NCS. It consists of needle electrodes (either monopolar or concentric) placed inside the muscle to record its potential at rest and during voluntary contraction. It records spontaneous electrical activities inside the denervated muscle at rest as deflections on an oscilloscope called positive sharp waves and fibrillation. Similarly, it records the muscle MUP during voluntary contraction. Such recordings help determine nerve injury duration, possible site, nature, and severity [34]. It also helps distinguish between neuropathic and myopathic causes of weakness [29].

Since the EMG study detects the location of nerve lesions, the etiological factors of nerve injury can be identified based on the location. Such etiological factors may be nerve block, tourniquet, patient positioning, or surgical causes if the lesion is found distal to the site of the block, tourniquet area, pressure points, or surgical dissection, respectively.

Timing of EMG study

When the electrical control over a muscle group is lost following injury to the innervating nerve, muscle fibers begin to discharge spontaneously. This process of development of spontaneous electrical activities in the denervated muscle takes about two to four weeks following nerve injury. Therefore, the EMG study is conducted two to four weeks after the injury [28]. After four weeks, the beginning of the reinnervation process can interfere with the EMG study. The positive EMG study performed before two to four weeks indicates pre-existing neuropathy, which can be important documentary evidence for medicolegal purposes following nerve injury.

Results

Positive EMG studies: It means the presence of fibrillations and sharp waves when the muscle is at rest and unstable MUPs on muscle contraction. It indicates the denervation of that particular muscle.

Negative EMG studies: It means the absence of fibrillations and sharp waves or the presence of stable MUPs. It indicates the absence of a nerve lesion or completed reinnervation in the muscle.

Limitations

As this procedure is associated with pain and performed without sedation, anesthesia, or analgesia, it causes discomfort to the patient while performing the test. Some patients may be unable to complete the test or may not agree to repeat the test if required.

Diagnosis of the type of nerve injury based on NCS/EMG

**Figure 4:**
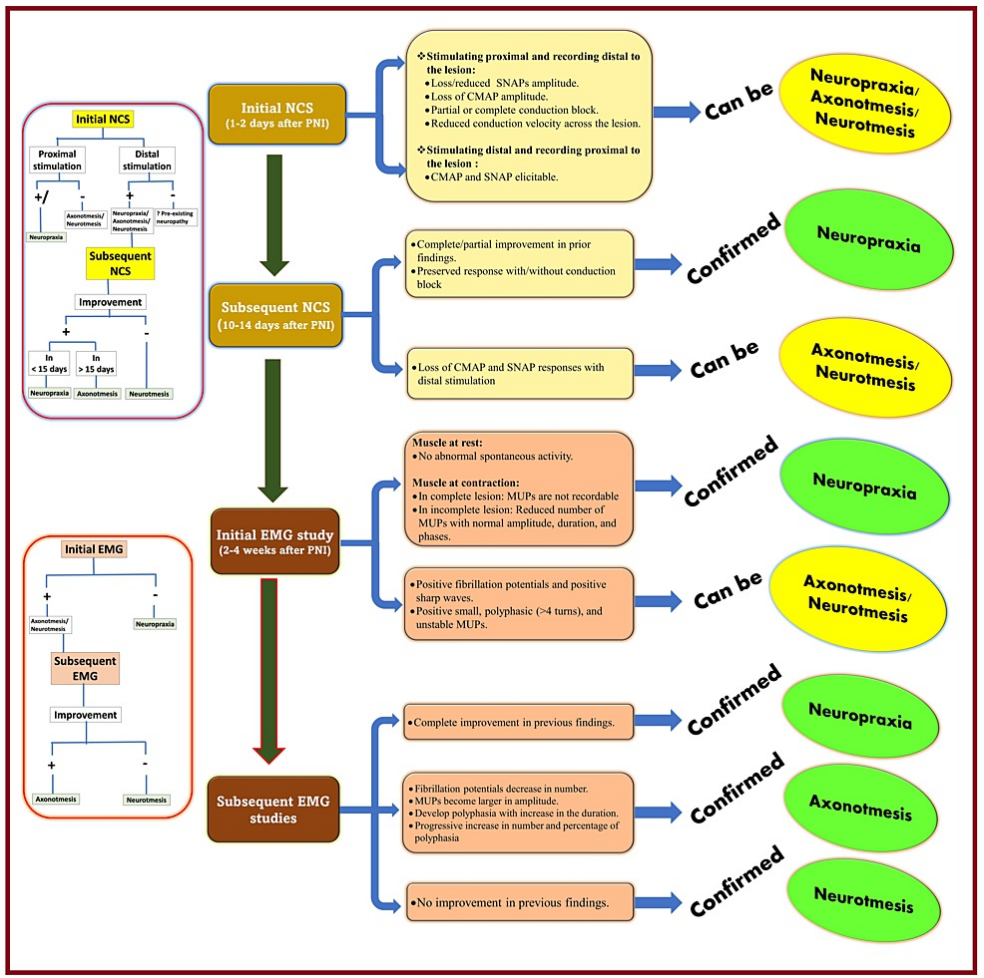
Diagnosis of types of nerve injury using sequential electrophysiological studies NCS: nerve conduction studies; PNI: peripheral nerve injury; SNAP: sensory nerve action potential; CMAP: compound muscle action potential; MUP: motor unit potential. Source: This figure was created by the first author (KS).

The timing of electrophysiological studies is very important to avoid misinterpretations when diagnosing nerve lesions. It depends on post-injury normal physiological time frames for degeneration, regeneration, remyelination, and reinnervation. Positive electrophysiological studies prior to the actual nerve injury suggest pre-existing neuropathies. Initial electrophysiological studies immediately after suspected nerve injuries help to detect and locate nerve lesions. At the same time, subsequent follow-up studies help to determine the type and progress of nerve lesions (Figure 4) [27-34].

As mentioned, the NCS detects nerve lesions, and the EMG study locates them. The timing of the NCS is one to two days, and the EMG study is two to four weeks after nerve injury. Following an injury, the motor and sensory axons remain excitable until 7-11 days (seven days for motor and 11 days for sensory axons) [35], and the Wallerian degeneration also sets in after 10-12 days. During this period, it becomes difficult to distinguish neuropraxia from axonotmesis. Therefore, NCS follow-up should be recommended 10-14 days after nerve injury [33]. In neuropraxia, stimulation of the distal portion of the nerve injury is normal. In contrast, CMAP and SNAP are absent when stimulated proximally or have reduced amplitude or conduction velocity. Subsequent NCS at 10-14 days may demonstrate preserved distal stimulation in neuropraxia. On the other hand, a lack of distal stimulation indicates neurotmesis or axonotmesis due to the onset of Wallarian degeneration. A follow-up study within a month shows an improvement in NCS values in neuropraxia as recovery in neuropraxia occurs within a week to a month. However, serial studies can only show an improvement in axonotmesis and no motor potential elicitation in neurotmesis.

EMG study records fibrillations and sharp waves in the denervated muscle when the muscle is at rest and MUP when the muscle is contracting. Regeneration and reinnervation processes can lead to a decrease in fibrillation and sharp waves [36] and an increase in the number, amplitude, duration, and percentage of polyphasia of MUP. The neuropraxia type of nerve injury is associated with the absence of abnormal spontaneous activities and an absent or reduced number of MUPs [28]. In neurotmesis and axonotmesis, the earliest changes are visible only after two to three weeks. They begin with abnormal spontaneous electrical activity and unstable MUPs due to the presence of regenerating sprouts [28]. During recovery, the MUP stabilizes, normalizes, and spontaneous electrical activities decrease.

Intraoperative electrophysiological studies

Intraoperative electrophysiological studies are also carried out during the surgical treatment of nerve injuries. It includes NCS, in which stimulating electrodes are placed on the nerve to be tested, and the nerve action potential is recorded using a suprathreshold stimulus [28,37] from the innervated muscle. Such studies help identify nerve injury and determine nerve continuity, help locate the site of the lesion, prevent surgical damage to the nerve by identifying the nerve in the surgical field, and help identify the nerve during neurolysis to avoid excessive resections and repairs. It also helps deal with the lesion in continuity that follows nerve injury, where supporting structures are preserved, but most nerve fibers are damaged [38].

Spontaneous or free-running EMG is often used as part of intraoperative neuromonitoring (Table 2) in the spine or neurological surgery. It helps to monitor selective nerve root function intraoperatively by displaying real-time recordings of peripheral muscles without stimulation [39,40]. It can help prevent postoperative radiculopathy during spine surgery. It is sensitive to nerve root irritation due to spinal cord or nerve root retraction, saline flushing, and manipulation during surgery. Neurotonic discharges in the form of spikes or bursts of activity are recorded from the selected muscle group due to traction, stretching, or compression of nerves during surgical procedures [39,40]. Such a recording indicates the proximity of the nerve roots. In contrast, trains of higher frequency and/or amplitude indicate a high probability of nerve injury.

**Table 2:** Common EMG recording sites by spinal levels EMG: Electromyography.

Spinal level	EMG site
C4	Supraspinatus.
C5	Deltoid and biceps.
C6	Biceps and wrist extensors.
C7	Triceps, wrist flexors.
	Finger extensors.
C8	Hand intrinsics.
	Finger flexors.
T1	Hand intrinsics.
T6-T12	Rectus abdominis.
L1	Iliopsoas.
L2	Adductor longus.
L3	Adductor longus.
	Vastus medialis.
L4	Vastus medialis.
	Vastus lateralis.
L5	Tendoachilis.
	Extensor hallucis longus.
S1	Medial gastrocnemius.
	Peroneus longus.
S2-S5	Perianal musculature.
	Urethral sphincter.

Treatment options for the nerve injury

Management of the patient with a nerve injury depends on the severity of the neurological symptoms, whether minor or major deficits [41]. The treatment goals in postoperative nerve injury include treating the cause, providing symptomatic relief, and reassuring and informing the patient simultaneously. Patients with minor deficits usually require reassurance, regular follow-ups, and symptomatic treatment due to the self-resolving nature of such types of injuries over a period of time [41]. However, patients with major deficits need a multidisciplinary approach to address the complexity of the symptoms with simultaneous regular assessments and symptomatic care [41]. Regardless of the type, the patient should be evaluated clinically to determine if a more serious or occult injury or correctable contributory condition is not being overlooked.

Among several potential causes, acute progressive causes of the nerve injury must be identified first during initial evaluation and examination. They need urgent treatment as they are reversible or treatable. Otherwise, it can lead to functional neurological deficits if left untreated. Such causes include restrictive plaster casts, compartment syndrome, hematoma, direct nerve trauma, traction, sutures, and screws/clips impinging the nerve. Further imaging studies and surgical intervention in the form of hematoma evacuation, compartment pressure release, or nerve repair can be needed to treat such causes.

Neuropraxic injuries require the wait-and-watch approach with conservative management and regular follow-ups to look for progression or improvement of symptoms. In comparison, severe injuries such as axonotmesis or neurotmesis require a neurosurgical consultation, imaging, and serial electrophysiological studies. Deficits of common entrapment sites are mostly neuropraxic, requiring a wait-and-watch approach with reassurance, analgesics, physiotherapy, and regular follow-up care. Lesions associated with pre-existing neuropathy can be diffuse or multifocal. It requires a bilateral examination and a document assessment to compare with the preoperative status. The treatment protocol is the same for the mild variant (a conservative approach) and the severe variant (imaging and neurological consultations).

Multimodal Analgesia

Appropriate pain management procedures have a wide spectrum, including anti-neuropathic pain medication (Table 3) [42,43], PNBs, neuromodulation, nerve repair, neurolysis, or spinal cord/deep brain stimulation. Pain associated with nerve injury or neuropathic pain differs from postoperative nociceptive pain. However, both neuropathic and nociceptive pain can overlap or coexist postoperatively. Neuropathic pain can be severe, excruciating, and stabbing with an electric shock-like feeling. It can be associated with tingling and/or altered sensation over the affected region, weakness or loss of motor power, dry and reddened skin due to disruption of sympathetic functions, or positive Tinels sign (gentle tapping on the nerve leading to a feeling of electric shock) [44,45]. Neuropathic pain management should focus on desensitization and normal use of the affected limb. No medication as such has been proven to regenerate the nerve, but many drugs can reduce inflammation, irritation, and firing and thus aid in nerve regeneration. Medications such as steroids can aid nerve recovery and regeneration by reducing inflammation [46]. Supplements such as methylcobalamin also support the nerve regeneration process [47]. Antidepressants and anxiolytics help patients manage the anxiety that accompanies their unwanted symptoms.

**Table 3:** Drug therapy for neuropathic or nerve injury-related pain NMDA: N-methyl-D-aspartate; GABA: gamma-aminobutyric acid; MOP: mu opioid peptide.

Drug type	Mechanism of action	Side effects
Tricyclic Antidepressants: (Amitriptyline, paroxetine, imipramine)	Blocking the reuptake of Serotonin and norepinephrine, blocking the NMDA receptor, blocking voltage-dependent sodium channels, and maintaining spinal cord GABA(B) receptor activity.	Decreased salivation, constipation, urinary hesitancy, blurred vision, orthostatic hypotension, sedation, cognitive impairment, and Torsade de pointes from prolongation of the Q-T interval (only in higher doses).
Anticonvulsants: (Gabapentin,pregabalin, carbamazepine,topiramate, levetiracetam, oxcarbazepine)	Directly or indirectly inhibit excitatory neurotransmitters, block neuronal calcium channels, and augment central nervous system inhibitory pathways by increasing GABA transmission.	Drowsiness, anxiety, visual disturbances, hypertension, and ataxia.
Nonsteroidal anti-inflammatory drugs: (Ibuprofen, naproxen, diclofenac, meloxicam, celecoxib, acetaminophen)	Inhibit the cyclooxygenases, which synthesize prostaglandins.	Gastrointestinal side effects (include indigestion and stomach ulcers), headache, drowsiness, dizziness, allergic reaction, and myocardial infarction or stroke risk.
Opioids: (Oxycodone, oxycontin, fentanyl patches)	Inhibition of neurotransmitter release from the primary afferent terminals in the spinal cord and activation of descending inhibitory controls in the midbrain.	Sedation, dizziness, constipation, and opioid tolerance and dependence.
Tramadol	Phenylpiperidine analog of codeine, weak agonist effect on MOP (μ) receptors, and norepinephrine and Serotonin reuptake inhibitor.	Lowers seizure threshold, nausea, low incidence of constipation, and minimum risk of addiction and abuse.
Capsaicin Ointment (0.025% - 0.075%)	Deplete the transmitter contents of small afferent fibers leading to degeneration and subsequent pain-generating ability.	Burning sensation before analgesia.

Multidisciplinary Approach

Early neurological consultation is essential in case of suspected or confirmed surgical nerve injury. It helps to set a baseline, which is good for prognostication, and determine the direction of evaluation and further care. In case of mild deficits such as neuropraxia, continued recommended conservative management can delay neurological consultation until one month. However, neurological consultation may be sought if neuropraxic injuries do not resolve within a month. Simultaneous physical and occupational therapy consultations help prevent contractures, muscular atrophy, and ongoing disability. Both have an important role in avoiding any secondary fracture or disuse atrophy in the muscle innervated by the injured nerve and maintaining the strength and tonicity of the particular muscle group during recovery. Psychological consultation helps patients develop coping mechanisms to manage pain in conjunction with other treatments [48]. Social services consultations help the patient engage in activities of daily living as early as possible.

## Conclusions

Aside from PNB, several possible causes need to be considered. The possibility of nerve injury in PNB should not deter refining RA practices or practicing safe RA techniques. Understanding patients and their comorbidities is always good before developing procedure-specific RA techniques. Multifactorial nerve injury requires a multidisciplinary approach that primarily includes reassuring, psychological counseling, multimodal analgesia, and neurological and occupational consultations. The first step is to reassure and counsel the patient. In this case, good communication is important. Also, initiating supportive care in the form of analgesics for pain, anti-neuropathic drugs, and steroids for other symptoms gives the patient psychological support and a sense of being helped. However, further research is needed in the form of comparative, analytical, diagnostic, and outcome measurement studies to accelerate the diagnosis and treatment of nerve injuries and attain optimal functional outcomes.

## References

[1] Urban MK, Urquhart B (1994). Evaluation of brachial plexus anesthesia for upper extremity surgery. Reg Anesth.

[2] Borgeat A, Ekatodramis G, Kalberer F, Benz C (2001). Acute and nonacute complications associated with interscalene block and shoulder surgery: a prospective study. Anesthesiology.

[3] Weyker PD, Webb CA, Pham TM (2016). Workup and Management of Persistent Neuralgia following Nerve Block. Case Rep Anesthesiol.

[4] Lalkhen AG, Bhatia K (2012). Perioperative peripheral nerve injuries. Continuing Educ Anaesth Critical Care Pain.

[5] (2023). Assessment of neurologic complications of regional anesthesia. https://www.nysora.com/topics/complications/assessment-neurologic-complications-regional-anesthesia/.

[6] Staff NP, Engelstad J, Klein CJ (2010). Post-surgical inflammatory neuropathy. Brain.

[7] Wani ML, Ahangar AG, Ganie FA, Wani SN, Wani NU (2012). Vascular injuries: trends in management. Trauma Mon.

[8] Timberlake GA, O'Connell RC, Kerstein MD (1986). Venous injury: to repair or ligate, the dilemma. J Vasc Surg.

[9] Bui MH, Hung DD, Vinh PQ, Hiep NH, Anh LL, Dinh TC (2019). Frequency and risk factor of lower-limb deep vein thrombosis after major orthopedic surgery in Vietnamese patients. Open Access Maced J Med Sci.

[10] Hadzic A (2006). Textbook of Regional Anesthesia and Acute Pain Management.

[11] Hwang JY (2017). Doppler ultrasonography of the lower extremity arteries: anatomy and scanning guidelines. Ultrasonography.

[12] Assir MZ, Das JM (2023). How to Localize Neurologic Lesions by Physical Examination. https://pubmed.ncbi.nlm.nih.gov/29630211/..

[13] Clark A, M Das J, Weisbrod LJ (2023). Trauma Neurological Exam. https://www.ncbi.nlm.nih.gov/books/NBK507915/.

[14] Kowalska B, Sudoł-Szopińska I (2013). Ultrasound assessment of selected peripheral nerve pathologies. Part III: Injuries and postoperative evaluation. J Ultrason.

[15] Alaqeel A, Alshomer F (2014). High resolution ultrasound in the evaluation and management of traumatic peripheral nerve injuries: review of the literature. Oman Med J.

[16] Padua L, Di Pasquale A, Liotta G (2013). Ultrasound as a useful tool in the diagnosis and management of traumatic nerve lesions. Clin Neurophysiol.

[17] Wijntjes J, Borchert A, van Alfen N (2020). Nerve ultrasound in traumatic and iatrogenic peripheral nerve injury. Diagnostics (Basel).

[18] Blanch XS, López AM, Carazo J, Hadzic A, Carrera A, Pomés J, Valls-Solé J (2009). Intraneural injection during nerve stimulator-guided sciatic nerve block at the popliteal fossa. Br J Anaesth.

[19] Koenig RW, Pedro MT, Heinen CP, Schmidt T, Richter HP, Antoniadis G, Kretschmer T (2009). High-resolution ultrasonography in evaluating peripheral nerve entrapment and trauma. Neurosurg Focus.

[20] Goel A, Knipe H, Omar Carrim Y (2023). Nerve injury classification (MRI). https://radiopaedia.org/articles/47058.

[21] Sneag DB, Zochowski KC, Tan ET (2021). MR neurography of peripheral nerve injury in the presence of orthopedic hardware: technical considerations. Radiology.

[22] Chhabra A, Ahlawat S, Belzberg A, Andreseik G (2014). Peripheral nerve injury grading simplified on MR neurography: Aas referenced to Seddon and Sunderland classifications. Indian J Radiol Imaging.

[23] Chhabra A, Andreisek G, Soldatos T, Wang KC, Flammang AJ, Belzberg AJ, Carrino JA (2011). MR neurography: past, present, and future. AJR Am J Roentgenol.

[24] Husarik DB, Saupe N, Pfirrmann CW, Jost B, Hodler J, Zanetti M (2009). Elbow nerves: MR findings in 60 asymptomatic subjects--normal anatomy, variants, and pitfalls. Radiology.

[25] Jarvik JG, Yuen E, Haynor DR (2002). MR nerve imaging in a prospective cohort of patients with suspected carpal tunnel syndrome. Neurology.

[26] Love S, Kalhorn S (2018). Utility of MRI after negative CT. Clinical Imaging of Spinal Trauma: A Case-Based Approach.

[27] Preston CD, Shapiro BE (2005). Electromyography and Neuromuscular Disorders. Electromyography and Neuromuscular Disorders.

[28] Kamble N, Shukla D, Bhat D (2019). Peripheral nerve injuries: electrophysiology for the neurosurgeon. Neurol India.

[29] Quan D, Bird SJ (1999). Nerve conduction studies and electromyography in the evaluation of peripheral nerve injuries. Univ Pennsylvania Ortho J.

[30] Kimura J, Machida M, Ishida T, Yamada T, Rodnitzky RL, Kudo Y, Suzuki S (1986). Relation between size of compound sensory or muscle action potentials, and length of nerve segment. Neurology.

[31] Watson JC (2017). Assessment of neurologic complications of regional anesthesia. Hadzic's Textbook of Regional Anesthesia and Acute Pain Management, 2e.

[32] Neal JM, Rathmell JP (2006). Complications in Regional Anesthesia and Pain Medicine.

[33] Parry GJ (1992). Electrodiagnostic studies in the evaluation of peripheral nerve and brachial plexus injuries. Neurol Clin.

[34] Preston CD, Shapiro BE (2005). Electromyography and Neuromuscular Disorders. 2nd ed..

[35] Chaudhry V, Cornblath DR (1992). Wallerian degeneration in human nerves: serial electrophysiological studies. Muscle Nerve.

[36] Kraft GH (1990). Fibrillation potential amplitude and muscle atrophy following peripheral nerve injury. Muscle Nerve.

[37] Shukla DP, Devi BI (2011). Management of the lesion in continuity: how important is the intraoperative electrophysiology? Treatment of peripheral nerve lesions. WFNS.

[38] Mavrogenis AF, Pavlakis K, Stamatoukou A (2008). Current treatment concepts for neuromas-in-continuity. Injury.

[39] Lall RR, Lall RR, Hauptman JS (2012). Intraoperative neurophysiological monitoring in spine surgery: indications, efficacy, and role of the preoperative checklist. Neurosurg Focus.

[40] Park JH, Hyun SJ (2015). Intraoperative neurophysiological monitoring in spinal surgery. World J Clin Cases.

[41] Deschner S, Borgeat A, Hadzic A (2007). Chapter 69. Neurologic complications of peripheral nerve blocks: mechanisms & management. NYSORA Textbook of Regional Anesthesia and Acute Pain Management.

[42] Cavalli E, Mammana S, Nicoletti F, Bramanti P, Mazzon E (2019). The neuropathic pain: an overview of the current treatment and future therapeutic approaches. Int J Immunopathol Pharmacol.

[43] Fornasari D (2017). Pharmacotherapy for neuropathic pain: a review. Pain Ther.

[44] Costigan M, Scholz J, Woolf CJ (2009). Neuropathic pain: a maladaptive response of the nervous system to damage. Annu Rev Neurosci.

[45] Campbell JN, Meyer RA (2006). Mechanisms of neuropathic pain. Neuron.

[46] Falvo E, Diviccaro S, Melcangi RC, Giatti S (2020). Physiopathological role of neuroactive steroids in the peripheral nervous system. Int J Mol Sci.

[47] Zhang M, Han W, Hu S, Xu H (2013). Methylcobalamin: a potential vitamin of pain killer. Neural Plast.

[48] Sturgeon JA (2014). Psychological therapies for the management of chronic pain. Psychol Res Behav Manag.

